# Supplementary material for: Patient Perspectives on Portal-Based Anxiety and Depression Screening in HIV Care: A Qualitative Study Using the Consolidated Framework for Implementation Research
Source: Int J Environ Res Public Health. 2024 May 28;21(6):692. doi: 10.3390/ijerph21060692 (PMC11203430; doi:10.3390/ijerph21060692)
Supplement: Supplementary file 1 [file ijerph-21-00692-s001.zip › ijerph-2969594-supplementary.pdf]

PORTAL HIV-A

Focus Group/One-On-One Interview Guide for Patients

Purpose

- Thank you for agreeing to participate and share your opinion about a new procedure that is being implemented in the clinic.
- This is an opportunity to voice your opinion and your feedback will inform the development of the new procedure.

Procedures

- At this discussion session, I will first ask you to fill out a brief demographic survey, if you have not already done so.
- Then we will have a discussion in which I will be asking questions about your thoughts and feelings about a new procedure to screen and monitor anxiety among those served by the clinic.
- You can opt to not answer any questions you choose, and your participation is completely voluntary.
- The discussion may take up to 90 minutes.
- The most important rule for this discussion is that everything you tell us morning/afternoon/tonight will be kept confidential. To protect your confidentiality, we will only audio record this meeting, you have been assigned a fake name, and we have disabled video for all participants.

General questions not about the portal

- What do you think about screening for anxiety at your HIV care visits?
- What do you think about screening for depression at your HIV care visits?
- What are your thoughts about being asked about anxiety or depression during HIV care appointments?
- Who should ask you questions about anxiety or depression during HIV care appointments?
- What ways would you prefer to be asked about anxiety or depression related to your HIV care?
- How is your mental health currently managed? Is it managed by the HIV clinic? If so, how so?

Intervention characteristics

- We are designing a system to screen and monitor anxiety and depression for PLWH using the patient portal (MyChart). Are you aware of the patient portal? How do you use it?

- We have been able to develop a similar workflow in the UCM General Internal Medicine Clinic. I'd like to walk you through the workflow and get your feedback on it. [Show PowerPoint figures and explains]. In this workflow, patients receive an invitation to complete assessments via the portal.
- What do you think of the invitation? How would you feel about getting it?
- The patient then can log in to their portal account and completes the assessment.
    - Over the last 2 weeks, how often have you been bothered by any of the following problems?
        - Little interest or pleasure in doing things
        - Feeling down, depressed, or hopeless
        - Feeling nervous, anxious or on edge
        - Not being able to stop or control worrying
- What do you think about the questions for depression? Would you feel comfortable answering them?
- What do you think about the questions for anxiety? Would you feel comfortable answering them?
- These results are automatically sent to their provider and stored to the patient's medical record. The patient's care team reviews the cases of patients who screened positive via the portal and provides follow-up care as needed.
- Who would you want to follow-up on the results if they were positive?
- Would you feel comfortable if it was a social worker, a nurse?
- What do you think about assessing anxiety and depression using the patient portal? What concerns do you have?
- What information would other patients need to know to increase the chance of them complete the anxiety and depression screening on the patient portal?
- How frequently do you think PLWH should be asked these anxiety and depression screening questions through the patient portal?
- How many reminders should be sent (for people who don't fill them out)? How often?
- How would you prefer to receive the message from the portal prompting you to complete the mental health screening? Would you feel comfortable answering these questions by phone, text, or in the waiting area?
- What types of interventions would you like to see in this clinic?
- What services or resources are you getting in the community pertaining to mental health care?

Outer setting

- How will using a patient portal meet your needs?
- What are the barriers of anxiety and depression screening using the patient portal?

Inner Setting

- Is there is strong need to measure anxiety and depression using the patient portal?

Characteristics of Individuals

- How do you feel about this method of assessing anxiety and depression in the HIV care clinic? Anticipation? Stress? Enthusiasm? Why?
- How confident are you that you will be able to use the patient portal? What gives you that level of confidence?

Process

- In general, when a person has been identified as having depression and/or anxiety symptoms via the patient portal some type of follow-up care and/or outreach is done. Who is the best person to reach out to this person? A social worker, nurse, provider, other?
- How often should this person reach out to the person?
- What type of resources and/or care should this person provide?

Wrap-up

- What do you think we should be asking you about to help with your healthcare?
- Is there anything else that you would like to share that I did not ask about?
